# Atypical Presentation of Traumatic Aortic Injury

**DOI:** 10.1155/2014/864301

**Published:** 2014-12-30

**Authors:** Andrew Fu Wah Ho, Tallie Wei-Lin Chua, Puneet Seth, Kenneth Boon Kiat Tan, Sohil Pothiawala

**Affiliations:** ^1^SingHealth Emergency Medicine Residency Program, Singapore Health Services, 167 Jalan Bukit Merah No. 17-10 Tower 5, Singapore 150167; ^2^Department of Emergency Medicine, Singapore General Hospital, Outram Road, Singapore 169608

## Abstract

*Background*. Blunt thoracic aorta injury (BAI) is second only to head injury as cause of mortality in blunt trauma. While most patients do not survive till arrival at the hospital, for the remainder, prompt diagnosis and treatment greatly improve outcomes. We report an atypical presentation of BAI, highlighting the diagnostic challenges of this condition in the emergency department. *Case Presentation*. A previously well 25-year-old male presented 15 hours after injury hemodynamically stable with delirium. There were no signs or symptoms suggestive of BAI. Sonography showed small bilateral pleural effusions. Chest radiograph showed a normal mediastinum. Eventually, CT demonstrated a contained distal aortic arch disruption. The patient underwent percutaneous endovascular thoracic aortic repair and recovered well. *Conclusion*. This catastrophic lesion may present with few reliable signs and symptoms; hence, a high index of suspicion is crucial for early diagnosis and definitive surgical management. This paper discusses the diagnostic utility of clinical features, injury mechanism, and radiographic modalities. Consideration of mechanism of injury, clinical features, and chest radiograph findings should prompt advanced chest imaging.

## 1. Background

High energy blunt chest trauma puts multiple structures at risk of injury, either via direct trauma or by rapid deceleration and other mechanisms. Blunt thoracic aorta injury (BAI) is second only to head injury as the leading cause of mortality in blunt trauma [1]. While a vast majority of patients with BAI do not survive until transport to the hospital, for the remainder, prompt diagnosis and treatment greatly improve outcomes [2].

We report an atypical presentation of BAI which highlights the challenges of diagnosing this condition in the emergency department.

## 2. Case Presentation

A previously well 25-year-old Malay male was transferred to our emergency department (ED) from a foreign hospital after being involved in a motor vehicle accident. Transfer notes and accounts from the family detailed that the accident occurred 15 hours prior to triage at our ED. The patient was the back passenger in a car travelling between 70 and 80 kilometers per hour. The driver swerved to avoid hitting an animal, thereafter colliding into the concrete curb, and came to a stop in the jungle. The driver walked out of the car and saw the door on the patient's side caved in with shattered glass. The patient was unconscious for a short while and later started speaking incoherently. The other passengers got away with an elbow fracture and minor injuries.

He was overseas then and was taken to a nearby hospital. While awaiting a computed tomogram (CT) scan of the abdomen and pelvis, the family decided to transfer him against medical advice to our ED in Singapore for further management.

During transfer, his vital signs remained stable. On arrival in our ED, he had normal vital signs, with a pulse rate of 76 beats per min, blood pressure of 125/58 mmHg, and oxygen saturation of 100% on room air. He was noted to be confused and did not respond to questions.

**Figure 1:**
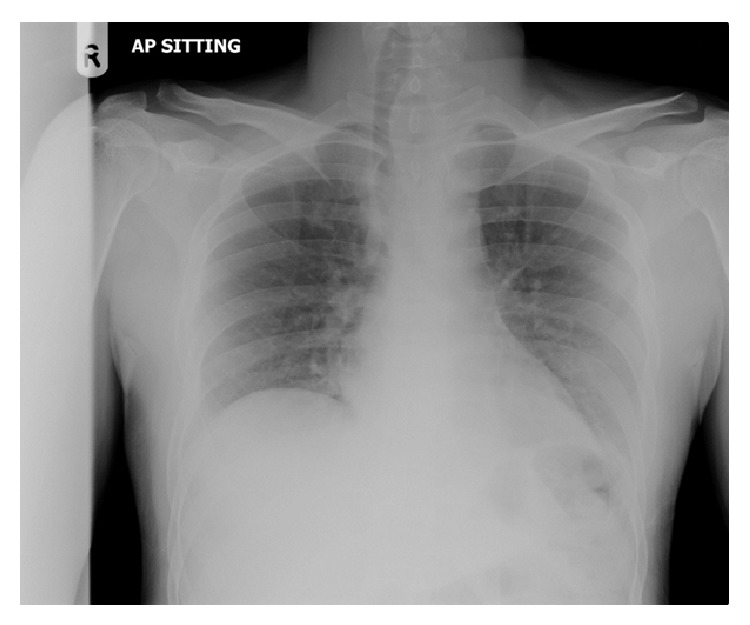
Chest radiograph showing tracheal deviation but otherwise unremarkable.

Auscultation of the chest was unremarkable. There were superficial bruises over the face and left arm, as well as deep lacerations over the right elbow, with no obvious communication with the joint space. There were no other obvious injuries found on physical examination. There were no external signs of injury to the chest. Extended focused assessment with sonography in trauma (E-FAST) showed minimal bilateral pleural effusions. The chest radiograph (Figure 1) showed only tracheal deviation to the right with apparent soft tissue in the left lower neck, which appeared to be related to the thyroid.

**Figure 2:**
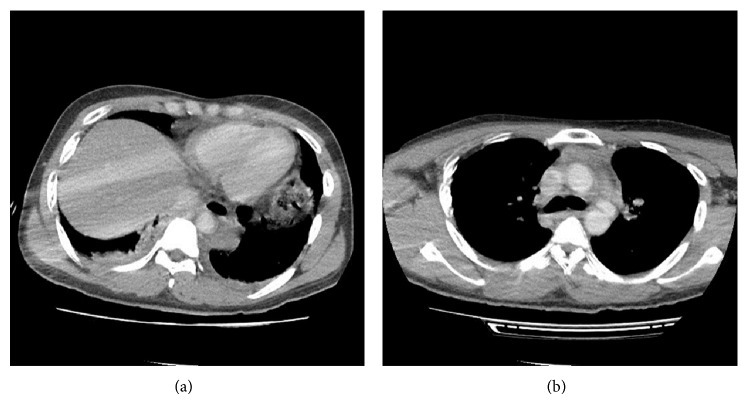
Computed tomogram showing (a) 2 × 1 cm contrast-filled outpouching at the anterolateral aspect of the distal aortic arch with (b) associated mediastinal fat stranding and crescentic soft tissue density along the descending thoracic aorta.

Our hospital trauma team was activated. CT brain was unremarkable. Decision was made to proceed with a CT of the chest, abdomen, and pelvis. The CT scan showed a 2 × 1 cm contrast-filled outpouching at the anterolateral aspect of the distal aortic arch (Figure 2(a)) with associated mediastinal fat stranding and crescentic soft tissue density along the descending thoracic aorta (Figure 2(b)). There was bilateral small dense pleural fluid. There was also a well-circumscribed 5.4 × 3.9 × 6.1 cm left lower neck mass likely arising from the thyroid. The trachea is displaced to the right with 50–60% luminal narrowing. There were possible hepatic lacerations and hyperdense pelvic fluid compatible with hemoperitoneum.

An urgent cardiothoracic consult was obtained. A CT aortogram was performed which demonstrated a traumatic aortic disruption with no active contrast extravasation. His blood pressure was controlled with IV labetalol and was transferred to the emergency operating room. He underwent a successful percutaneous thoracic endovascular aortic repair and also a laparotomy and peritoneal washout which found hemoperitoneum with a stellate liver laceration on liver capsule segment 5/6.

Recovery was uneventful and the patient was discharged functionally and neurologically well. Followup reveals that he returned to gainful employment.

## 3. Discussion

80–85% of patients with blunt thoracic aorta injury die before reaching the hospital [3, 4]. In some patients who survive transport to hospital, the rupture is contained by the adventitia or mediastinal structures, as in the reported patient. These patients usually sustain secondary aortic rupture within 24 hours [5]. Our patient presented 15 hours after injury and may have suffered a secondary aortic rupture.

Observation studies found some clinical features to suggest the presence of BAI [6]. These include hypotension, upper extremity hypertension, bilateral lower extremity pulse deficit, and initial chest tube output greater than 750 mL of blood. Patients with BAI were also found to have greater incidence of other significant injuries, with one retrospective study finding 31% with concomitant major head injury and 29% with major abdominal injury [7].

However, the clinical features are unreliable as their absence cannot exclude the presence of BAI. 30% of patients with BAI have no external signs of chest trauma while 75% will have rib fractures which draw attention away from concomitant intrathoracic injury [5].

Given that clinical features are unreliable, the injury mechanisms should already raise suspicion of BAI. High-energy blunt trauma involving rapid deceleration is at risk. Motor vehicle crashes account for 74% of BAI in one study [8]. The rest are contributed by falls from height and crushing injuries to the chest. An autopsy case review in 142 patients with BAI proposed that the pathogenesis of aortic rupture involves a lateral oblique compression impact to the chest, which causes thoracic mediastinal structures to shift and deflect the aortic arch, resulting in severe shearing and stretching at the isthmus [9].

Abnormal chest radiograph findings, especially widening of the mediastinum, should prompt advanced chest imaging. However, even in the absence of chest radiograph findings, a suspicion of BAI based on injury mechanism and clinical features should similarly prompt chest imaging. Current technology favors a CT scan as the modality of choice, as opposed to transesophageal echocardiogram or CT angiography [10].

This case highlights an atypical presentation without any suggestion from clinical features and chest radiograph findings. A clear history of the injury mechanism was not available. Early diagnosis was especially valuable in this case as there was no other significant injury which would complicate surgical management and recovery.

## 4. Conclusion

This case highlights that BAI can be remarkably occult. This catastrophic lesion can present with few reliable signs and symptoms and hence early diagnosis is contingent on high index of suspicion to facilitate early definitive surgical management. Consideration of mechanism of injury and clinical features as well as chest radiograph findings should prompt advanced chest imaging.
